# Breast Cancer Patient-Derived Scaffolds Can Expose Unique Individual Cancer Progressing Properties of the Cancer Microenvironment Associated with Clinical Characteristics

**DOI:** 10.3390/cancers14092172

**Published:** 2022-04-26

**Authors:** Elena Garre, Anna Gustafsson, Maria Carmen Leiva, Joakim Håkansson, Anders Ståhlberg, Anikó Kovács, Göran Landberg

**Affiliations:** 1 Department of Laboratory Medicine, Institute of Biomedicine, Sahlgrenska Academy, Sahlgrenska Center for Cancer Research, University of Gothenburg, SE-41390 Gothenburg, Sweden; anna.gustafsson.3@gu.se (A.G.); maria.leiva@randstad.se (M.C.L.); anders.stahlberg@gu.se (A.S.); 2 Department of Clinical Pathology, Sahlgrenska University Hospital, SE-41345 Gothenburg, Sweden; aniko.kovacs@vgregion.se; 3 Division Material and Production, Department of Methodology, Textile and Medical Device, Unit for Biological Function, RISE Research Institutes of Sweden, SE-50115 Borås, Sweden; joakim.hakansson@ri.se; 4 Department of Laboratory Medicine, Institute of Biomedicine, University of Gothenburg, SE-40530 Gothenburg, Sweden; 5 Wallenberg Center for Molecular and Translational Medicine, University of Gothenburg, SE-41390 Gothenburg, Sweden; 6 Department of Clinical Genetics and Genomics, Sahlgrenska University Hospital, Västra Götaland Regional Council, SE-41345 Gothenburg, Sweden

**Keywords:** translational research, tumor microenvironment, cancer stem cells, patient-derived scaffolds, breast cancer

## Abstract

**Simple Summary:**

Despite huge progress in cancer diagnostics and medicine we still lack optimal cancer treatments for patients with aggressive diseases. This problem can be influenced by the biological heterogeneity of cancer cells as well as poorly understood cancer promoting effects of the cancer microenvironment being an important part of the cancer niche. In this study we have specifically monitored the activity of the cancer microenvironment in breast cancer patients using cell-free scaffolds repopulated with reporter cancer cells sensing the activity of the patient environment. The data show that scaffold induced changes in epithelial-mesenchymal transition and pluripotency markers were linked to clinical and prognostic properties of the original cancer and the information was even more precise when matching estrogen receptor status in our system. The findings highlight that patient-derived scaffolds uncover important information about varying malignant promoting properties in the cancer niche and can be used as a complementary diagnostic tool.

**Abstract:**

Breast cancer is a heterogeneous disease in terms of cellular and structural composition, and besides acquired aggressive properties in the cancer cell population, the surrounding tumor microenvironment can affect disease progression and clinical behaviours. To specifically decode the clinical relevance of the cancer promoting effects of individual tumor microenvironments, we performed a comprehensive test of 110 breast cancer samples using a recently established *in vivo*-like 3D cell culture platform based on patient-derived scaffolds (PDSs). Cell-free PDSs were recellularized with three breast cancer cell lines and adaptation to the different patient-based microenvironments was monitored by quantitative PCR. Substantial variability in gene expression between individual PDS cultures from different patients was observed, as well as between different cell lines. Interestingly, specific gene expression changes in the PDS cultures were significantly linked to prognostic features and clinical information from the original cancer. This link was even more pronounced when ERα-status of cell lines and PDSs matched. The results support that PDSs cultures, including a cancer cell line of relevant origin, can monitor the activity of the tumor microenvironment and reveal unique information about the malignancy-inducing properties of the individual cancer niche and serve as a future complementary diagnostic tool for breast cancer.

## 1. Introduction

Breast cancer is a highly heterogeneous disease, where structure and composition vary greatly between individual tumors. Histological and molecular characteristics allow for the classification into intrinsic breast cancer subtypes which are clinically used to assess treatment approaches [1]. However, the tumor microenvironment (TME) is an often overlooked parameter in cancer diagnostics, where the focus is set on the malignant cells despite the link between the TME and cancer-related processes, such as treatment resistance, tumor progression and metastasis formation [2]. The tumor microenvironment is composed of a variety of cell types, including immune cells, endothelial cells and fibroblasts. These cells and their surrounding extracellular matrix (ECM) interact through signalling, as well as via dynamic and mechanical interactions [3]. The ECM has previously merely been considered as a mechanical support for the cancer cells, but several aspects of the dysfunctional disposition of the ECM have now been associated with clinically relevant properties such as drug resistance, tumor growth and invasive capacity [4]. A large amount of the current knowledge about cellular and molecular tumour biology was provided by studies in 2D cultures [5], despite that these simplified cultures cannot recreate the complexity of tissue architectures and cellular interactions occurring *in vivo*. Epithelial-mesenchymal transition (EMT) is a fundamental cellular process linked to metastasis that is poorly reproduced in monolayer cultures because of the lack of support from ECM and protein-protein binding [6,7]. Similarly, the tumor initiating subpopulation of cancer stem cells (CSC) thrives in specific niches in the TME, influencing resistance to cancer treatments and thereby potentially leading to therapeutic failure and disease recurrences [8]. Because of the importance of the entire milieu for cancer cells, there is a large focus on the development of optimal 3D culture methods that can reproduce and mimic the intricate TME. This will produce more relevant experimental model systems that can increase the understanding of key processes in cancer progression and also increase the success rate of drug development using more *in vivo*-like growth platforms in discovery programs.

Matrigel and synthetic matrixes are among the most popular substances to reproduce the tumor ECM in vitro allowing for the study of cell-cell and cell-ECM interactions in a 3D environment, but with the drawback of lacking human-specific compositions or structures [9]. On the other hand, 3D bioprints have the ability to engineer the tissues to emulate specific tumor characteristics, while keeping high reproducibility [9,10]. Nevertheless, all these methods lack the ability to represent the disease diversity that is present in patient-derived models. Organoids derived from primary cancer specimens are a promising tool for the study of cancer cell-specific properties in personalized medicines, but lack the information provided by the interaction from patient specific ECM and stromal cellular components [11]. Recently, the use of decellularized patient-derived cancer tissues have emerged as an in vivo-like 3D culture technique that can recapitulate the characteristics of the human TME. Cell-free tissues maintain most of the ECM structure and tumor composition, and have successfully been used in several types of malignancies including colorectal cancer, lung cancer, glioblastoma, and breast cancer [12,13,14,15,16,17,18,19].

Our research group has developed a patient-derived scaffold (PDS) method based on decellularized primary breast cancers that are repopulated with standard breast cancer cell lines in order to study the influence of specific TMEs on cellular behaviours. An advantage with the comprehensive PDS model is that not only structural ECM proteins are included in the cell-free scaffolds used as growth platforms, but also patient specific soluble factors linked to processes such as secretion, stress response, immunomodulation and drug resistance are present, originating from different cell types in the cancer niche [18,20]. Our previous studies have shown that PDS growth induced substantial but varying phenotype enrichment of the cancer stem cell population, with a parallel increase in EMT features and a decrease in proliferation [18]. Cancer cells cultured in PDSs further showed an increased drug resistance and sometimes disparate drug responses compared to 2D cultures [21,22]. Interestingly, when analysing the protein composition of cell-free PDS we also observed a variability in the proteomic composition between patients and associations between protein presences and clinical features as well as gene expression modulations of PDS-cultured cancer cells [18,20].

In the current study, we addressed how the different origins of cancer cell lines interacted and adapted to the tumor microenvironment presented by the PDSs based on the intrinsic characteristics of the cancer cells and the tumor from the PDSs were generated, using a large breast cancer cohort with available clinic-pathological data and analysing gene expression changes in three different breast cancer cell lines adapted to the different microenvironments. The results support that PDSs indeed reveal unique information linking clinical properties to specific tumor microenvironments in the form of cell-free scaffolds.

## 2. Materials and Methods

### 2.1. Patient Material

Frozen breast cancer tissues were collected from Sahlgrenska University Hospital Breast Biobank (Gothenburg, Sweden). Samples were obtained from 1992 to 1999 and followed up for disease-free survival (DFS) until 2012. A cohort of 110 patients were included in this study and all of them underwent surgery, either total or partial mastectomy, without neoadjuvant therapy (Supplementary Table S1).

### 2.2. Patient-Derived Scaffold Generation and Cryosectioning

Frozen breast tumors were decellularized as described [18,22]. In brief, tumors were decellularized in two rounds of lysis buffer containing 5 mM EDTA, 0.1% sodium dodecyl sulfate, 0.4 mM phenylmethylsulfonyl fluoride (PMSF) and 0.02% sodium azide (all Sigma-Aldrich, St. Louis, MO, USA) in water for 6 h, followed by a rinse step with water supplemented with sodium azide, EDTA and PMSF at the same concentrations for 15 min. During the following 72 h, PDSs were washed with H_2_O and, thereafter, for 24 h with phosphate buffered saline solution (PBS; Medicago) to remove cellular debris. Decellularization was performed at 37 °C and agitation at 175 rpm (Incu-ShakerTM 10 L, Benchmark). Patient-derived scaffolds were then placed in a storage solution containing sodium azide, EDTA and PBS at 4 °C to preserve the tissue until usage. Patient-derived scaffolds were mechanically made into even sections using biopsy punch needles of Ø 6 mm. Samples were then snap-frozen in liquid nitrogen and sectioned to 150 µm thin slices using CM3050 S (Leica) cryotome. The PDS slices were then sterilised in PBS supplemented with 0.1% peracetic acid (Sigma-Aldrich) for 1 h at room temperature, followed by 24 h wash with PBS containing 1% Antibiotic-Antimycotic (Thermo Fisher Scientific, Waltham, MA, USA), at 37 °C and 175 rpm.

### 2.3. Cell Culture Methods

MCF7, T-47D and MDA-MB-231 cell lines (ATCC, Manassas, VA, USA; HTB-22™, HTB-133™ and HTB-26™ respectively) were cultured in accordance with ATCC recommendations. MCF7 cells were cultured in Dulbecco’s modified Eagle’s medium (DMEM) supplemented with 10% fetal bovine serum, 1% penicillin and streptomycin, 1% L-glutamine, 1% Antibiotic-Antimycotic (all Thermo Fisher Scientific) and 1% MEM non-essential amino acids (Sigma-Aldrich); while T-47D and MDA-MB-231 were cultured in RPMI-1640 medium supplemented with 10% fetal bovine serum, 1% streptomycin and penicillin, 1% L-glutamine, 1% Sodium Pyruvate (all Thermo Fisher Scientific) and 1% Antibiotic-Antimycotic. All cultures were performed in humidified chambers at 37 °C with 5% CO_2_, and cell lines were confirmed as mycoplasma- free (Mycoplasma PCR Detection Kit, Applied Biological Materials Inc., Richmond, BC, Canada).

Patient-derived scaffold slices were cultured with MCF7, T-47D or MDA-MB-231. 3 × 10^5^ cells were added to each PDS slice in 48-well plates containing 0.5 mL cell line specific media. Patient-derived scaffolds were transferred to a new plate with fresh media after 24 h and every four to seven days, depending on the cell growth rate, and incubation was continued for 21 days. Regular 2D cultures in plastic dishes were performed in parallel to the PDS cultures and used as reference for gene expression adaptation analyses.

### 2.4. Gene Expression Analysis

Cells grown in PDSs and in 2D cultures were harvested in 350 µL of RLT buffer (Qiagen, Hilden, Germany) and stored at −80 °C until RNA extraction. Samples were homogenized with stainless steel beads in TissueLyser II (Qiagen), 5 min × 2 at 25 Hrz, centrifuged at 14,000 rpm for 3 min, and supernatants were transferred to new tubes. RNA was extracted using a RNeasy Micro kit in QIAcube, including DNAse digestion (all Qiagen). RNA concentration was measured using NanoDrop (Thermo Fisher Scientific) and RNA quality was randomly assessed using a 5400 Fragment analyser HS RNA Kit (15NT) (DNF-472).

Subsequently, 100–400 ng of RNA were transcribed using a GrandScript cDNA synthesis kit, including RNA Spike II (all TATAA Biocenter) for RNA stability control, in T100 Thermal Cycler (Bio-Rad) using a temperature profile of 25 °C for 5 min, 42 °C for 30 min, 85 °C for 5 min and cooling at 4 °C. cDNA was diluted 1:5 or 1:6 in RNAse-free water (Thermo Fisher Scientific) and added to 1xSYBR GrandMaster Mix (TATAA Biocenter) and 400 nM primer mix (Supplementary Table S2) for a real-time quantitative PCR (qPCR) reaction in a CFX384 Touch Real-time PCR Detection System (Bio-Rad). The temperature profile used was 95 °C for 2 min, 35–50 cycles of amplification at 95 °C for 5 s, 60 °C for 20 s and 70 °C for 20 s, and melting curve analysis from 65 °C to 95 °C with 0.5 °C/s increments. Cycle of quantification (Cq) values were determined by the second derivative maximum method with the CFX Manager Software v.3.1 (Bio-Rad). Data pre-processing was performed with GenEx (MultiID). Cq values were normalized to reference genes identified with the NormFinder algoritm, transformed to relative quantities to 2D samples of the same cell line and converted to log_2_. All experiments were conducted in accordance with the MIQE guidelines [23].

### 2.5. Statistical Analyses

Principal component and heatmap analyses were performed in GenEx (MultiId). Statistical analyses were performed in SPSS statistics v.25 (IBM). Experimental data are presented as median ± SD, and data dispersion was calculated by inter-quartile range (IQR). Mann-Whitney U and Kruskall-Wallis statistical tests were performed for assessment of clinico-pathological and molecular parameters. Correlations between gene expressions were analyzed using Spearman’s correlation coefficients. The Kaplan–Meier method was used to estimate disease-free survival (DFS) using log-rank comparisons in different gene expression strata divided by median, first quartile (25%) or third quartile (75%). Univariate cox proportional hazards regression was used to estimate hazard ratio (HR) and confidence interval (CI) 95% for each gene. Cox regression was also used to identify independent prognostic markers in multivariable analyses (MVA) using DFS, defined as time from diagnosis until regional, local or distant recurrence or breast cancer-specific death, and covariates were grade (1 and 2 versus 3), ERα-status, age, tumor size and lymph node metastasis, and one gene at a time were presented as a continuous variable. In addition, subgroups including only PDSs from ERα-positive or ERα-negative tumors were independently analyzed. *p*-values < 0.05 were considered significant. Benjamini-Hochberg corrections were assessed for multiple testing at α = 0.15, and adjusted *p*-values (q-values) were calculated with an FDR online calculator (SDM).

## 3. Results

### 3.1. Patient-Derived Scaffold Cultures Modulate Gene Expression Profiles in Patient- and Cancer Cell Line-Dependent Manners

To delineate the impact of unique tumor microenvironments from patients on standardized cancer cell lines, 110 patient-derived scaffolds (PDSs) were generated from biobanked primary breast tumors with available clinical follow-up data, including 89 ERα-positive, 19 ERα-negative tumors and 2 ERα status unknown (detailed patient characteristics in Supplementary Table S1). Cell-free sections from each PDS were repopulated with either the ERα-positive cell lines MCF7 (*n* = 110) or T-47D (*n* = 110), or the ERα-negative cell line MDA-MB-231 (*n* = 84). After 21 days of growth, gene expression of key markers associated with cancer-related processes were analysed by qPCR, including pluripotency (*POU5F1*, *NANOG*, *SOX2* and *NEAT1*), epithelial-mesenchymal transition (EMT) (*SNAI1*, *SNAI2*, *FOSL1*, *VIM* and *CDH2*), proliferation (*MKI67*, *CCNA2* and *CCNB2*), and breast cancer stem cell (CSC) (*CD44*, *ALDHIA3* and *ABCG2*) related genes.

**Figure 1:**
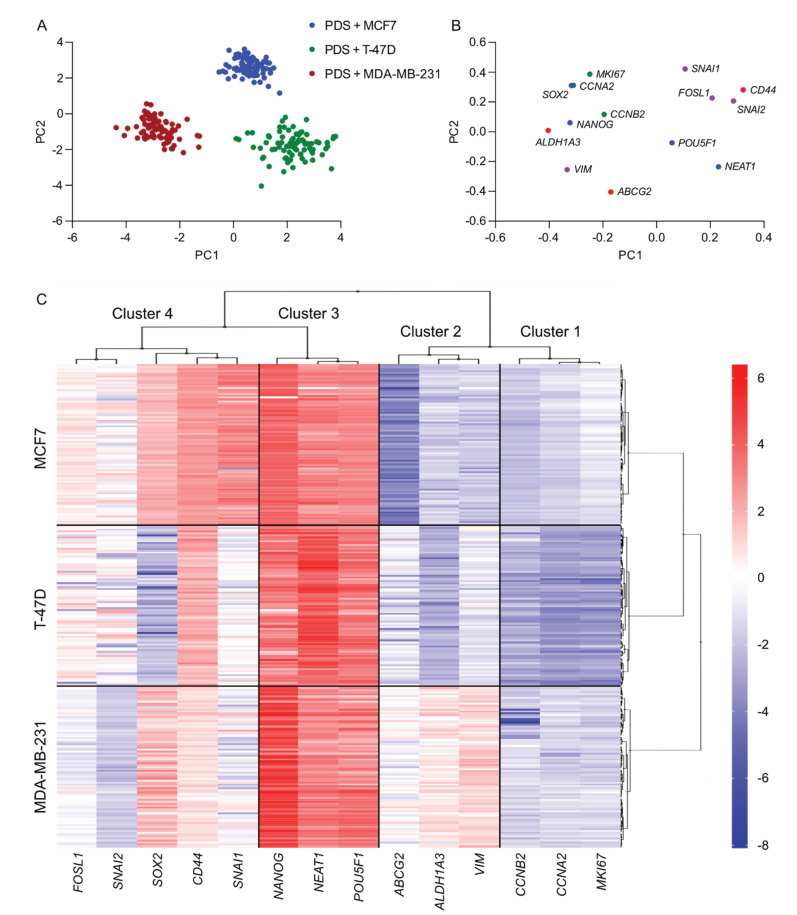
Changes in gene expression in patient-derived scaffold cultures cluster in cell line-dependent manner. Unsupervised analyses were applied to the expression of 14 genes associated with relevant cancer processes in 84 patient-derived scaffolds (PDSs) cultured with three different cell lines. (**A**) Principal component analysis (PCA) scores showing the PDSs cultured with MCF7 (blue), T-47D (green), and MDA-MB-231 (red). (**B**) PCA loading plot illustrating the genes’ contribution to the separation in the plot (A). Colour code markers: green, proliferation; blue, pluripotency; red, breast cancer stem cells (CSC); and purple, epithelial-mesenchymal transition (EMT). (**C**) Heatmap visualization grouping the genes (columns) and PDSs (rows) using average linkage as clustering method and Euclidean distances as the distance measure. Gene expression in PDSs is expressed in log2-scale, and relative to 2D controls for each cell line.

Gene expression changes induced by the different patient-based microenvironments in the three adapting cancer cell lines were analysed using unsupervised learning algorithms in the 84 overlapping PDS cultures (*n* = 67 ERα-positive cancers, *n* = 15 ERα-negative cancers, *n* = 2 ERα unknown) (Figure 1). Principal component analysis (PCA) identified three distinct clusters, one containing the PDSs cultured with MCF7, another with T-47D, and the last one with MDA-MB-231 (Figure 1A,B). Since the gene expression changes were normalized to the corresponding 2D cultures, the distinct separation between the three clusters indicated that the cell lines were influenced differently by the unique PDS microenvironments. Interestingly, different degrees of scattering within each cluster were observed, where PDSs cultured with MCF7 showed a higher uniformity in response to the microenvironments, while T-47D samples were more scattered, suggesting a more dynamic response of the cells to their adjacent surroundings.

A heatmap analysis revealed the genes responsible for the observed separation between cell lines and 4 different gene clusters, as indicated in Figure 1C. Cluster 1 consisted of the proliferation genes *MKI67*, *CCNA2* and *CCNB2* and showed a general downregulation in the three cell lines. Cluster 3 showed a similar trend for all PDSs and cell lines and was defined by the upregulation of the three pluripotency markers (*POU5F1*, *NEAT* and *NANOG*). In contrast, Clusters 2 and 4 presented a mix of EMT and CSC markers and the pluripotency marker *SOX2*, and showed pronounced variability between cell lines, as well as between individual PDSs.

**Figure 2:**
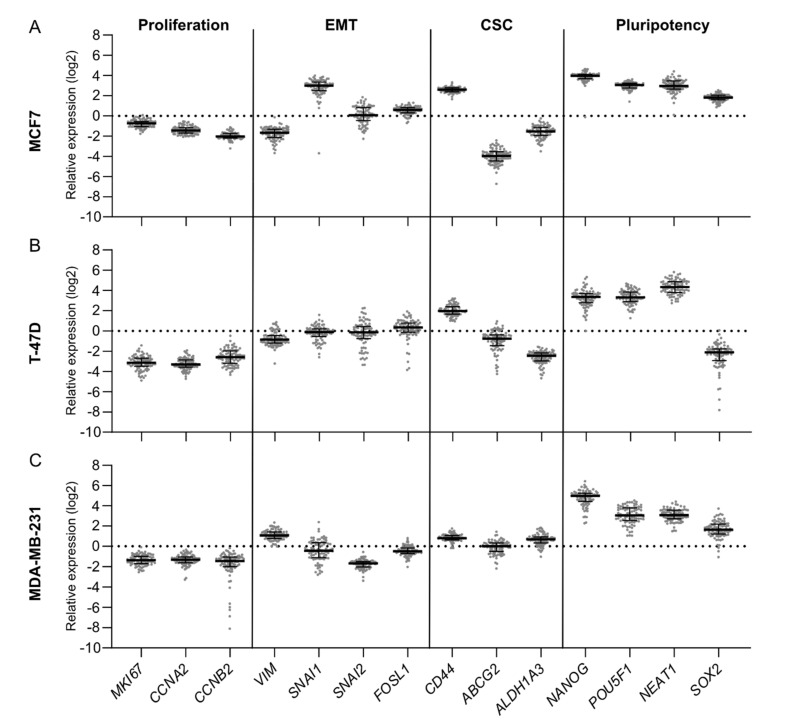
Gene expression fingerprints of patient-derived scaffolds cultured with different cell lines reveal tissue-specific variations. Individual dots represent relative gene expression of an individual patient-derived scaffold (PDS) (*n* = 84) grown with either (**A**) MCF7, (**B**) T-47D or (**C**) MBA-MD-231 to gene expression of the same cell line cultured in 2D. Genes related to proliferation, epithelial-mesenchymal transition (EMT), breast cancer stem cells (CSC) and pluripotency were included in the gene panel. Median and interquartile range (IQR) represented by errors bars are plotted. The IQR for each gene was calculated as measurement of the inter-PDS variability (Supplementary Table S3).

Visualization of the gene expression data as “fingerprints” for each cell line (Figure 2) also highlighted that the major differences between the cell lines adapted to PDSs involved EMT and CSC-related genes. When focusing on the EMT markers’ expression in the PDS cultures, the ERα-positive cell lines MCF7 and T-47D were characterized by a downregulation of *VIM*, which was mostly pronounced in MCF7 cells, as well as exhibiting minor changes of *SNAI2*; but showed distinct upregulation of *SNAI1* in MCF7 cells but with no changes in T-47D cells (Figure 2A,B). In contrast to the ERα-positive cell lines, PDS-adapted MDA-MB-231 cells displayed increased *VIM* expression and downregulation of *SNAI2* (Figure 2C). For the CSC markers, both ERα-positive cell lines showed similar expression changes in *CD44* and *ALDH1A3* genes but with a substantial drop in *ABCG2* expression in MCF7 cells only. The ERα-negative cell line MDA-MB-231 showed a modest upregulation of the breast CSC markers. Typical for PDS cultured T-47D cells was a distinct downregulation of the pluripotency marker *SOX2* in contrast to the upregulation in the other two cell lines in parallel to the other three pluripotency markers. The results highlight that despite general changes induced by the PDSs in all cell lines tested, there were also more specific changes induced by the PDSs depending on the cancer cell lines that were adapting to the changed microenvironments.

The dynamic range of gene expression changes for the PDS cultures of each gene illustrated in Figure 2 could also be substantiated by calculations of the interquartile range (IQR) of inter-PDS variability (Supplementary Table S3). These data confirmed that the majority of scattering of gene expression changes due to PDS cultures was observed for the EMT and pluripotency-related genes, and that the inter-PDS variability was more evident in PDSs cultured with T-47D cells, as previously illustrated by the PCA.

### 3.2. Patient-Derived Scaffold-Induced Gene Expression Changes Were Associated with Clinico-Pathological Data and Disease Progression of the Original Tumor

To further substantiate if the induced inter-PDS variability in gene expression was due to the preservation of intrinsic factors in the PDSs linked to clinical characteristics of the original cancer, we evaluated the associations between changes in gene expression and clinico-pathological features such as grade, ERα-status, PR (progesterone receptor)-status and metastasis in lymph nodes (LN) (Table 1, Supplementary Table S4). Interestingly, several gene expression changes in the ERα-positive cell lines MCF7 and T-47D adapting to PDSs were significantly associated with clinical properties of the original breast cancers, whereas similar analyses using MDA-MB-231 cells did not produce any statistically significant associations.

**Figure 3:**
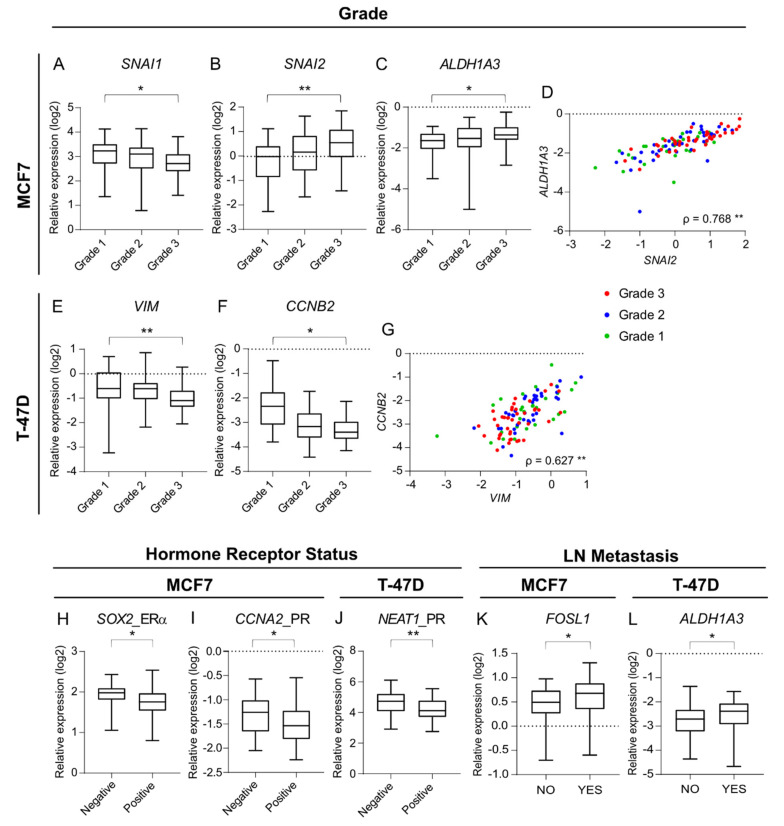
Correlations between patient-derived scaffolds-induced expression of key markers in MCF7 and T-47D cells associate with cancer-related processes and clinico-pathological variables of the original tumor. (**A**–**C**,**E**,**F**) Boxplots (min to max) representing the median and the spread of the patient-derived scaffold (PDS)-induced expression values for the genes showing significant association to the clinical variable grade, in MCF7 (**A**–**C**) and T-47D cells (**E**,**F**). (**D**,**G**) Scatter plots illustrating the correlation between *ALDH1A3* versus *SNAI2* gene expressions in MCF7 for each PDS (**D**) and the correlation between *CCNB2* versus *VIM* gene expressions in T-47D for each PDS (**G**), where colours denote the histological grade of the original tumor. (**H**–**L**) Boxplots (min to max) representing the median and the spread of the PDS-induced expression values for the genes showing significant association to the clinical variables progesterone receptor status (PR), estrogen receptor (ERα) status and presence of lymph node (LN) metastasis in MCF7 and T-47D cells (* *p*-value < 0.05, ** *p*-value < 0.01). Mann-Whitney U and Kruskall-Wallis statistical tests were performed for assessment of clinical variables (detailed information in Table 1). In the scatter plots, Spearman’s correlation coefficients (ρ) and the significance (** *p*-value < 0.01) are indicated (detailed information in Supplementary Figure S1 and Supplementary Table S5).

Among the clinical variables analysed, histological tumor grade showed the strongest link to the PDS-dependent expression changes (Table 1, Figure 3). Patient-derived scaffolds from high grade tumors induced changes in the expression of the EMT markers *SNAI1* and *SNAI2* in MCF7 cells but in opposite directions, producing lower levels of *SNAI1* (*p* = 0.015) and higher levels of *SNAI2* (*p* = 0.010) (Figure 3A,B). High expression of the breast CSC gene *ALDH1A3* (*p* = 0.030) was also associated with high tumor grade in PDS-grown MCF7 cells (Figure 3C). There was a further positive correlation between PDS-induced *ALDH1A3* and *SNAI2* expressions (*p* < 0.001, ρ = 0.768) (Figure 3D, Supplementary Figure S1, Supplementary Table S5). For T-47D cells, PDSs from high grade tumors induced lower expression of the EMT marker *VIM* (*p* = 0.004) (Figure 3E). The expression of *VIM* was further positively correlated with the proliferation marker *CCNB2* (*p* < 0.001, ρ = 0.627) which also was decreased in high grade tumors (*p* <0.001) (Figure 3F,G, Supplementary Figure S1, Supplementary Table S5).

Other strong associations were observed between the differential expression of several markers and the presence of hormone receptors, or the presence of lymph node metastasis in the original cancers. When growing MCF7 cells in PDSs generated from tumors lacking the ERα receptor, expression showed an increased expression of the pluripotency marker *SOX2* (*p* = 0.017), whereas PDSs from PR-positive tumors induced lower expression of the proliferation marker *CCNA2* (*p* = 0.032) (Figure 3H,I). Also, a significant link between the presence of the progesterone receptor in the original tumor and the expression of the pluripotency marker *NEAT1* in T-47D cells growing in PDSs was observed; where PDSs from PR-positive tumors induced a significant lower expression of this gene (*p* < 0.001), compared to the PDSs derived from tumors lacking the PR (Figure 3J). On the other hand, PDSs generated from breast cancers with associated lymph nodes (LN) metastasis were characterized by higher expressions of the EMT marker *FOSL1* (*p* = 0.047) and the breast CSC-related gene *ALDH1A3* (*p =* 0.024), when MCF7 or T-47D cell lines were used as reporters, respectively (Figure 3K,L).

**Figure 4:**
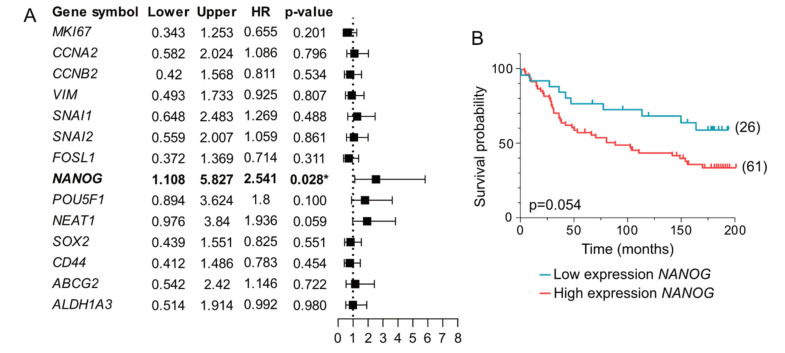
The expression of *NANOG* in MCF7 cells cultured in patient-derived scaffolds presents prognostic value. (**A**) Forest plot of the association between the expressions of individual genes in MCF7 cells growing in 84 patient-derived scaffolds (PDSs) and disease-free survival (DFS). Multivariable analyses (MVA) for each gene including ERα-status, grade, age, tumor size and lymph node metastasis was conducted, and HR (Cox proportional hazards regression), 95% confidence interval (lower and upper) and the corresponding *p*-value were calculated and plotted. (**B**) Kaplan-Meier plot illustrating the relationship between *NANOG* expression in MCF7 cells growing in PDSs and DFS (blue, low expression; red, high expression). A log-rank statistical test was applied to compare survival in different strata (M, median; Q1, first quartile; Q3, third quartile), the best cut-off and the *p*-value are shown (detailed information in Supplementary Table S7). (* *p*-value < 0.05).

To determine if the varying characteristics of the cancer microenvironments were associated with disease recurrences of patient’s cancers, we evaluated the relationship between gene expression changes induced by the PDS cultures and disease-free survival (DFS) for the patients included in the study. Interestingly, multivariable analyses (MVA) showed independent prognostic value for the pluripotency marker *NANOG* in PDSs cultured with MCF7 cells (HR, 2.541; 95% CI, 1.108–5.827; *p* = 0.028) after adjustment for ERα-status, grade, age, tumor size and lymph node metastasis (Figure 4A, Supplementary Table S6). The association between the high expression of *NANOG* and poor prognosis was corroborated in a Kaplan-Meier analysis stratifying patients based on low and high *NANOG* expression in PDSs (*p* = 0.054) (Figure 4B, Supplementary Table S7). Also, *NEAT1* and *POUF51* genes followed the same trend as *NANOG* in the MVA (Figure 4A), indicating that the pluripotency increasing capacity of the cancer microenvironments was associated with genuine cancer aggressiveness and disease recurrences in the patients.

### 3.3. The Concordance in ERα-Status between Patient-Derived Scaffolds and the Adapting Cancer Cell Line Strengthened the Link between PDS-Dependent Gene Expression Changes and Intrinsic Characteristics of the Original Cancer

The ERα-status is a key biomarker for breast cancer subtyping that strongly affects the tumor microenvironment’s characteristics [24]. To further identify potential links between gene expression changes in the PDS cultures and clinical features of the tumors, the ERα-status of both the adapting cell line and the tumor from which the PDS was generated was aligned.

Cultures of the ERα-positive cell line MCF7 growing in ERα-positive PDSs strengthened the previously observed associations described above between gene expression and clinico-pathological variables (Table 1, Supplementary Table S4), such as an increased expression of *SNAI2* (*p* = 0.005) and *ALDH1A3* (*p* = 0.014) in high grade cancer. Moreover, matching the ERα-status for the PDSs and cell line also uncovered new associations. High grade ERα-positive tumors were characterized by inducing lower expression of *FOSL1* in MCF7 cells (*p* = 0.025). The pluripotency marker *NEAT1* further showed higher expression in MCF7 cells grown in PDSs from patients with poor survival in MVA, when the ERα-positive group was analysed separately from the ERα-negative (HR, 2.289; 95% CI, 1.003–5.226; *p* = 0.049 and HR, 2.704; 95% CI, 0.833–8.770; *p* = 0.098, respectively) (Supplementary Figure S2, Supplementary Table S6).

**Figure 5:**
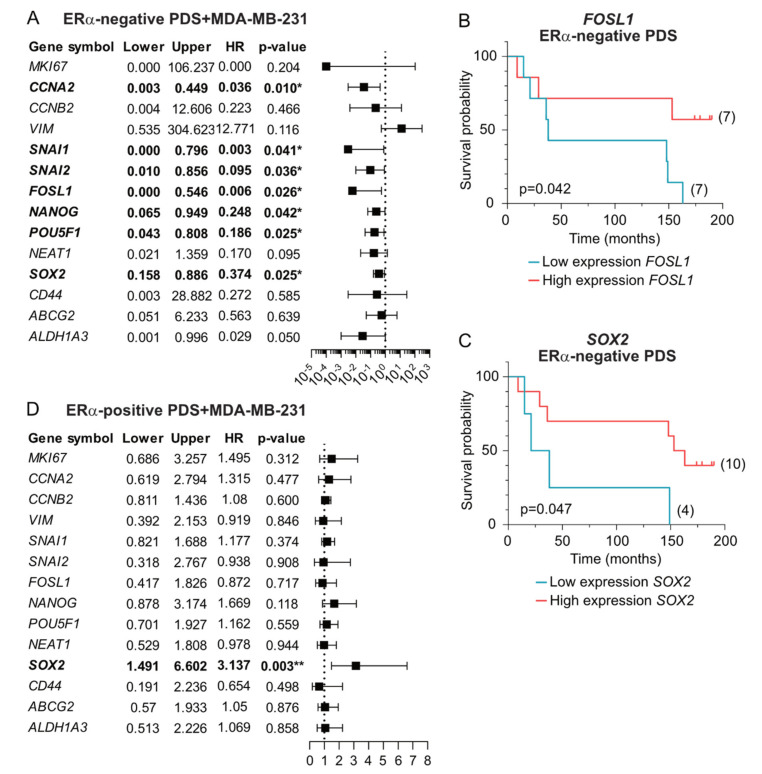
Induction of lower expression of several markers in ERα-negative MDA-MB-231 cells by growing in ERα-negative patient-derived scaffolds are related to the disease-free time of the donor patients. (**A**,**D**) Forest plots of the association between the expression of the individual genes in MDA-MB-231 growing in 14 ERα-negative patient-derived scaffolds (PDSs) (**A**) or 53 ERα-positive PDSs (**D**) and disease-free survival (DFS) of the patients whose tumors were used to generate the PDSs. Multivariable analyses (MVA) adjusted by ERα-status, grade, age, tumor size and lymph node metastasis was conducted for each gene, and Cox proportional hazards regression (HR), 95% confidence interval (lower and upper) and the corresponding *p*-value were calculated and plotted. (**B**,**C**) Kaplan-Meier plots illustrating the relationship between expression of genes *FOSL1* or *SOX2* expression in MDA-MB-231 cells growing in ERα-negative PDSs and DFS (blue, low expression; red, high expression). A log-rank statistical test was applied to compare survival in different strata (M, median; Q1, first quartile; Q3, third quartile), and the best cut-off and *p*-values are shown (detailed information in Supplementary Table S7). * *p*-value < 0.05, ** *p*-value < 0.01.

Novel associations with DFS were uncovered when the ERα-negative group of PDSs was cultured with the ERα-negative cell line MDA-MB-231 (Figure 5, Supplementary Table S6). Both univariate (HR, 0.130; 95% CI, 0.018–0.953; *p* = 0.045) and MVA including the clinical factors grade, age, size and lymph node metastasis (HR, 0.006; 95% CI, 0.000–0.546; *p* = 0.026) showed that PDSs inducing lower expression of the EMT marker *FOSL1* was significantly correlated with a poor prognosis of the patients in this subgroup, which could further be supported in Kaplan-Meier analyses (Figure 5A,B). MVA also revealed the independent prognostic value of the EMT markers *SNAI1* (HR, 0.003; 95% CI, 0.000–0.796; *p* = 0.041) and *SNAI2* (HR, 0.095; 95% CI, 0.010–0.856; *p* = 0.036), the proliferation marker *CCNA2* (HR, 0.036; 95% CI, 0.003–0.449; *p* = 0.010), and the pluripotency markers *NANOG* (HR, 0.248; 95% CI, 0.065–0.949; *p* = 0.042), *POU5F1* (HR, 0.186; 95% CI, 0.043–0.808; *p* = 0.025) and *SOX2* (HR, 0.374; 95% CI, 0.158–0.886; *p* = 0.025), all of them showing that lower expression of these markers induced by decellularized tumors was associated to poor survival (Figure 5A). None of these genes, except for *SOX2* (Figure 5C), were nevertheless statistically significant in Kaplan-Meier analyses using these small cohorts (Supplementary Figure S2, Supplementary Table S7). Interestingly, the *SOX2* gene showed a duality as a prognostic marker, since a higher expression was associated to poor prognosis in ERα-positive PDSs grown with MDA-MB-231 (HR, 3.3137; 95% CI, 1.491–6.602; *p* = 0.003) (Figure 5D), contrary to the observations in the ERα-negative subgroup, suggesting indeed that the interactions between cell lines and the surrounding microenvironment are highly influenced by the PDS’ innate ERα characteristics.

## 4. Discussion

Numerous studies have shown that mutual interactions between tumor cells and the surrounding microenvironment that includes different cell types and the ECM as structural support, influence cancer progression and malignant features [25]. To better represent this complexity in vitro, the scientific community strives to develop 3D-based culture systems that can recapitulate the characteristics of the human tumor microenvironment. However, most of the established models lack the patient-specific features responsible for tumor heterogeneity, such as structural support or patient-specific components [26,27,28].

We have therefore developed a method that is based on patient-derived scaffolds (PDSs) from decellularized breast cancer samples that can support cell growth and influence the adapting reporter cancer cells, thereby monitoring the activity of the patient specific cancer microenvironment [18]. Our previous studies suggest that the PDS system retains the variability between patients provided by the unique characteristics of the TMEs [18,21,22]. In the current study, we confirmed the PDS’ ability to preserve individual microenvironments based on the original cancer features as supported by the results generated from 110 biobanked breast cancers. Available patient follow-up data, including histopathological and clinical characteristics, further allowed studies of associations between PDS-induced gene expression changes and the clinical features. Moreover, to clarify the importance of the subtype of the used cancer cell line in the PDS system, three different breast cancer cell lines were used to repopulate the PDSs, and the results suggested that a concordance between the ERα-status of the original cancer and the cancer cell line further increased the sensitivity of the measurements and monitoring of the adaptation to the cancer microenvironment. Importantly, although the chosen cancer cell line influenced the system, we observed defined adaption of the cancer cells by the individual PDS microenvironment.

Despite clear transcriptional changes in cells towards a less proliferative and more pluripotent phenotype for the three cancer cell lines cultured in PDS compared to 2D growth, cell line-specific PDS variations were also presented as illustrated by unsupervised clustering analyses. The results suggest that the PDS model in general enriched the cancer stem cell features, but with varying levels and marker genes depending on the cancer cell line used, which is in agreement with previously described differential metabolism and transcriptional regulations for these cell lines in functional assays for CSC enrichment [29,30]. Although MCF7 and T-47D are both ERα-positive luminal cell lines, they have several genetic and phenotypic differences that potentially influence the adaptation to the various cancer microenvironments [31,32]. The observation that MCF7 cells in the PDS-model displayed more widespread gene changes compared to the other cell lines is in line with earlier studies identifying the co-existence of distinct subpopulations with different grades of CSC and differentiated phenotypes for MCF7 cells in CSC-enriched 2D cultures compared to the transcriptional response in T-47D cells [29]. As previously reported [18], MDA-MB-231 cancer cells in PDS cultures were highly infiltrative and showed different histological differentiation stages while inducing fewer transcriptional changes for the selected marker genes. This may in part be explained by the basal-mesenchymal phenotype of this cell line and innate infiltrative capacity, masking the changes in differentiation that can be observed in the other two cell lines [29,33].

Importantly, several of the gene changes in cells triggered by the growth in PDSs were associated with clinico-pathological data of the original cancers, indicating that the PDSs recapitulated individual and unique information from the patient TME that could be decoded using the PDS model. This inter-PDS variability was particularly identified by differential expression of the EMT markers. A reciprocal influence between EMT processes and the tumor ECM is well known [34], and an increase in the ECM stiffness can also induce mesenchymal behaviours in tumor cells [35]. This is mainly mediated through the Wnt signalling pathway which is involved in the stabilization and upregulation of EMT regulators, such as Snail and Slug [36,37,38], supporting that the PDS structure variability may induce differential expression of those genes. In addition, several studies have shown that EMT profiles are associated with certain clinico-pathological characteristics such as histological grade and tumor subtype [39]. Gene expression analyses in breast cancer have reported increased *SNAI2* expression in high grade tumors, while *SNAI1* showed a reduced expression [40], similar to our observations in MCF7 cells cultured in PDSs. The increased expression of the breast cancer stem cell (CSC) marker *ALDH1A3* in PDSs from high grade breast cancers is also in line with several earlier studies done in tumor tissue [41,42,43,44]. These observations were strengthened by a strong positive correlation between the expression of *SNAI2* and *ALDH1A3* in MCF7 cells growing in PDSs, supporting the co-activation of EMT and CSC processes and that culturing cells in PDSs can decode features in the cancer microenvironment in aggressive breast cancer [45]. In addition to the EMT markers, other marker genes also modified their expression depending on the unique characteristics of the used PDSs. For instance, *ALDH1A3* expression in T-47D cells growing in PDSs was associated with the presence of lymph node metastasis [42], and the prevalence of high expression of the pluripotency markers *SOX2* and *NANOG* in MCF7 cells cultured in PDSs was linked to the absence of the ERα in the original tumor and low DSF, respectively, in agreement with previous observations in tumor tissue [46,47]. The presented results suggest that the PDS model has potential to provide patient-relevant prognostic data and information regarding aggressive behaviours. An example is the EMT gene *FOSL1* that has a critical role in cell migration and invasion, and that in primary breast cancer shows increased copy number and mRNA overexpression; it has now been observed to be associated with lymph node metastases in the PDS model [48].

In the cancer niche, there is an interplay between the cancer cells and the surrounding microenvironment which is mainly mediated by secreted molecules, such as cytokines and chemokines, which will influence and reprogram the surrounding cells [34,49,50,51]. Previous proteomic characterization of cell-free PDSs [18,20] identified, apart from structural proteins related to the ECM, numerous secreted molecules originating from cancer cells and other cells present in the original tumor stroma. Moreover, the protein composition varied between different PDSs and was also associated with the clinical parameters of tumor grade and Ki67-levels. In line with a variation in PDS composition linked to cancer cell properties, the presented results suggest that the selection of a cancer cell line for the PDS cultures having closer resemblance to the original cancer subtype and features would recognize and therefore also respond and adapt in a more relevant manner to the influence of the microenvironments provided by the PDSs. Generally, the ERα-positive MCF7 cells sensed and reproduced some of the intrinsic original cancer features better than the two other cell lines tested when the entire PDS cohort was analysed. However, most of the associations between expression changes in MCF7 cells and the clinical variables were strengthened in the ERα-positive PDS subgroup, indicating that by harmonizing the given TME with the reporter cells, the imprinted information in the PDSs could be more optimally decoded.

Since the majority of the ERα-negative breast cancer samples included in our cohort were also PR-negative and high grade [24,52,53], there was a limited capacity to find any association between expression of marker genes and those clinical variables, even when using the ERα-negative cell line MDA-MB-231. However, when analysing ERα-negative PDSs and other clinical variables, we observed several associations between marker genes and disease-free survival, with an important overrepresentation of EMT (*SNAI1*, *SNAI2* and *FOSL1*) and pluripotency markers (*NANOG*, *POU5F1* and *SOX2*). Most of these markers are crucial factors to produce pluripotent stem cells, and their expression in basal-like tumors have previously been linked to poor survival [54,55]. Interestingly, the expression of *SOX2* in MDA-MB-231 cells growing in both ERα-negative and ERα-positive PDSs when analysed independently was associated with patient poor survival but in opposite directions. These results indicate that the specific composition of the TME may act as a mediator of cancer cell plasticity, inducing different gene expression reprograming to allow the adaptation to the surroundings. However, future experiments including larger ERα-negative tumor cohorts needs to be conducted in order to verify these data.

## 5. Conclusions

Our current results support that PDS provide a unique tool to understand how cellular and molecular interactions within the tumor microenvironment modulate clinical aggressiveness and relevant tumor biological features, suggesting that the PDS model can be a useful complementary diagnostic tool. Previous studies have also demonstrated that the PDSs model can be used for “out-of the patients” drug tests and to evaluate the influence of the cancer microenvironment on the responses [21,22]. The collective data suggest that the PDS model can provide prognostic and treatment predictive insights, thereby contributing to optimized personalized medicine.

## 6. Patents

The patient-derived scaffold approach and data are patent pending.

## Figures and Tables

**Table 1:** Induced gene expression by PDS cultures is linked to clinico-pathological characteristics of the original tumor. Table shows *p*-values when the entire PDSs cohort was analysed together (all), and subdivided by ERα-status (ERα-, ERα-negative; ERα+, ERα-positive). Clinico-pathological features analysed: grade, ERα-status, PR (progesterone receptor)-status and metastasis in lymph nodes (LN). Data was analysed using SPSS statistics (IBM). Non-parametric Mann-Whitney U and Kruskall-Wallis statistical test were performed for assessment of clinico-pathological variables relationship with individual gene expression in PDSs. *p*-value < 0.05 were considered significant (* *p*-value < 0.05, ** *p*-value < 0.01). Significant *p*-values after Benjamini-Hochberg corrections (^§^ α = 0.15) are indicated. Detailed information in Supplementary Table S4.

			All				ER+			ER−		
			Grade	ERα	PR	LN	Grade	PR	LN	Grade	PR	LN
MCF7	Proliferation	*MKI67*	0.893	0.135	0.075	0.721	0.632	0.538	0.495	0.955	0.068	0.421
		*CCNA2*	0.933	0.300	0.032 *	0.560	0.607	0.196	0.975	0.737	0.035 *	0.080
		*CCNB2*	0.321	0.925	0.214	0.112	0.109	0.188	0.353	0.263	0.261	0.083
	EMT	*VIM*	0.291	0.624	0.703	0.604	0.304	0.400	0.525	0.434	0.888	0.573
		*SNAI1*	0.015 *^§^	0.132	0.241	0.547	0.019 *^§^	0.641	0.231	0.502	0.888	0.237
		*SNAI2*	0.01 *^§^	0.760	0.153	0.945	0.005 **^§^	0.204	0.800	1.000	0.206	0.633
		*FOSL1*	0.052	0.487	0.170	0.047 *	0.025 *^§^	0.269	0.134	0.695	0.888	0.474
	Pluripotency	*NANOG*	0.679	0.960	0.359	0.769	0.712	0.211	0.481	1.000	0.399	0.965
		*POU5F1*	0.236	0.970	0.725	0.511	0.141	0.578	0.299	0.371	0.673	0.408
		*NEAT1*	0.174	0.682	0.457	0.148	0.120	0.575	0.135	0.146	0.673	0.762
		*SOX2*	0.571	0.017 *	0.492	0.715	0.438	0.952	0.721	0.655	0.160	0.947
	BCSC	*CD44*	0.108	0.345	0.966	0.201	0.131	0.700	0.436	0.867	0.261	0.237
		*ABCG2*	0.140	0.759	0.226	0.754	0.074	0.370	0.316	0.911	0.035 *	0.327
		*ALDH1A3*	0.03 *^§^	0.874	0.528	0.833	0.014 *^§^	0.627	0.875	0.955	0.574	0.395
T-47D	Proliferation	*MKI67*	0.260	0.258	0.118	0.609	0.451	0.322	0.330	0.01 *^§^	0.673	0.633
		*CCNA2*	0.342	0.627	0.416	0.781	0.626	0.749	0.616	0.034 *	0.261	0.829
		*CCNB2*	0.024 *	0.360	0.637	0.858	0.064	0.357	0.837	0.434	0.261	0.744
	EMT	*VIM*	0.004 **^§^	0.113	0.262	0.424	0.026 *	0.490	0.333	0.314	0.574	0.829
		*SNAI1*	0.865	0.524	0.893	0.117	0.797	0.947	0.157	0.823	0.261	0.515
		*SNAI2*	0.734	0.618	0.888	0.530	0.796	0.884	0.374	0.602		1.000
		*FOSL1*	0.705	0.211	0.056	0.320	0.930	0.015 *^§^	0.889	0.399	0.136	0.036 *
	Pluripotency	*NANOG*	0.427	0.668	0.579	0.671	0.306	0.941	0.386	0.678	0.655	0.673
		*POU5F1*	0.264	0.390	0.092	0.075	0.429	0.256	0.046 *	0.314	0.122	0.897
		*NEAT1*	0.198	0.859	0.001 **^§^	0.086	0.098	0.005 **^§^	0.083	0.911	0.049 *	0.897
		*SOX2*	0.303	0.950	0.811	0.956	0.316	0.696	0.868	1.000	0.655	0.321
	BCSC	*CD44*	0.062	0.137	0.070	0.590	0.185	0.230	0.988	0.737	0.779	0.146
		*ABCG2*	0.476	0.546	0.638	0.431	0.418	0.911	0.402	0.146	0.325	0.897
		*ALDH1A3*	0.328	0.063	0.132	0.024 *	0.348	0.324	0.034 *	0.219	0.261	0.460
MDA-MB-231	Proliferation	*MKI67*	0.875	0.131	0.078	0.855	0.975	0.352	0.426	0.676	0.187	0.559
		*CCNA2*	0.383	0.089	0.176	0.599	0.672	0.512	0.511	0.552	0.923	0.779
		*CCNB2*	0.307	0.769	0.383	0.979	0.219	0.197	0.813	0.381	0.791	0.710
	EMT	*VIM*	0.274	0.186	0.770	0.285	0.423	0.259	0.125	0.305	1.000	0.620
		*SNAI1*	0.512	0.559	0.345	0.730	0.378	0.157	0.505	0.305	0.923	0.620
		*SNAI2*	0.712	0.511	0.166	0.691	0.451	0.435	0.320	0.933	0.264	0.535
		*FOSL1*	0.825	0.719	0.962	0.255	0.771	0.763	0.426	0.933	0.923	0.710
	Pluripotency	*NANOG*	0.846	0.489	0.929	0.878	0.919	0.941	0.511	0.800	0.198	0.259
		*POU5F1*	0.503	0.579	0.514	0.783	0.540	0.710	0.324	0.933	0.549	0.318
		*NEAT1*	0.972	0.927	0.098	0.669	0.994	0.102	0.921	0.476	0.132	0.128
		*SOX2*	0.690	0.960	0.138	0.213	0.715	0.107	0.148	0.476	0.549	0.902
	BCSC	*CD44*	0.605	0.105	0.369	0.846	0.671	0.787	0.833	0.790	1.000	0.740
		*ABCG2*	0.598	0.633	0.778	0.359	0.648	0.823	0.328	0.933	0.264	0.902
		*ALDH1A3*	0.156	0.612	0.478	0.818	0.284	0.435	0.640	0.371	0.429	0.831

## Data Availability

The data presented in this study are available in the Supplementary Material and on request from the corresponding authors.

## Supplementary Materials

The following supporting information can be downloaded at: https://www.mdpi.com/article/10.3390/cancers14092172/s1, Figure S1: Spearman’s correlation heatmaps; Figure S2: Kaplan-Meier plots illustrating the relationship between gene expression and DFS; Table S1: Clinical information; Table S2: Primers; Table S3: Interquartile range; Table S4: Associations between changes in gene expression and clinico-pathological features; Table S5: Spearman’s correlation; Table S6: Cox regression; Table S7: Kaplan Meyer cutoffs for disease-free survival.
