# Supplementary material for: Breast Cancer Patient-Derived Scaffolds Can Expose Unique Individual Cancer Progressing Properties of the Cancer Microenvironment Associated with Clinical Characteristics
Source: Cancers (Basel). 2022 Apr 26;14(9):2172. doi: 10.3390/cancers14092172 (PMC9103124; doi:10.3390/cancers14092172)
Supplement: Supplementary file 1 [file cancers-14-02172-s001.zip › Supp Figures_Garre_2022.pdf]

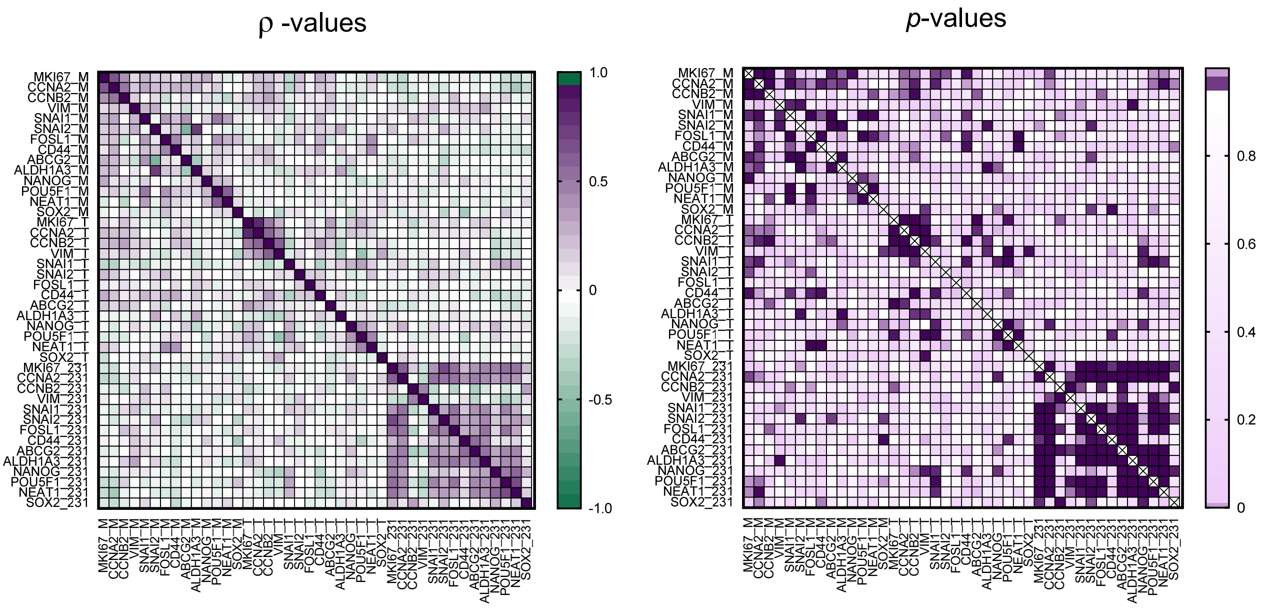

Supplementary Figure S1. **Spearman's correlation heatmaps.** Heatmap plotting the Spearman's correlation coefficients (ρ-values) and *p*-values between all the analysed genes and the three cell lines included in this study. (M, MCF7; T, T-47D; 231, MDA-MB-231). (Detailed information in Supp. Table S5)

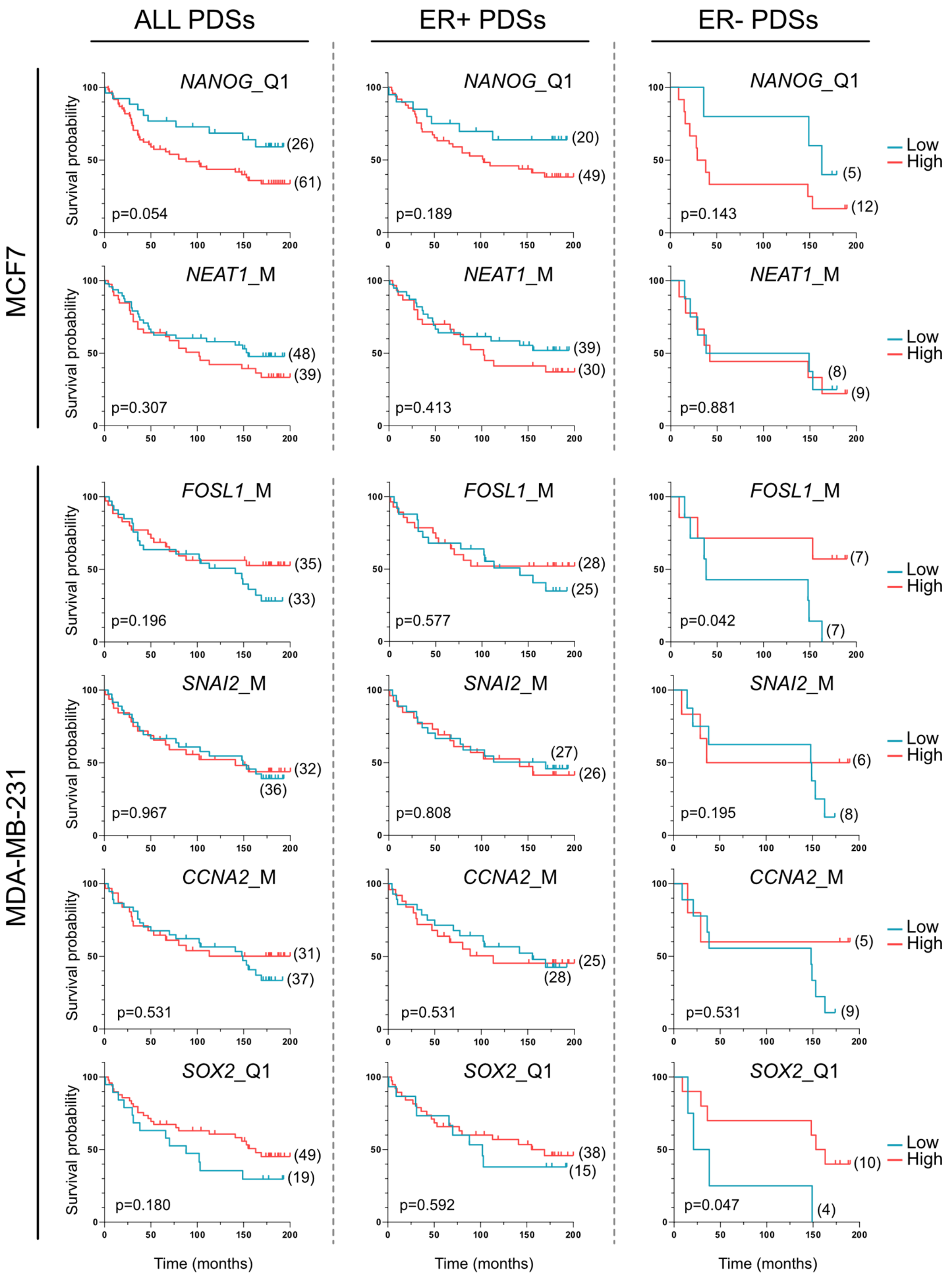

Supplementary Figure S2. Kaplan-Meier plots illustrating the relationship between gene expression and disease-free survival (DFS) of patients whose tumors were used to generate the patient-derived scaffolds PDSs (blue, low expression; red, high expression), for the genes that were significant in the multivariable analyses. The number of patients is indicated within brackets in each graph. Log-rank statistical test was applied to compare survival in different strata (M, median; Q1, first quartile; Q3, third quartile), the best cut-off and the *P*-value are shown (detailed information in Supp. Table S7). *p*-values <0.05 were considered significant.
